# MiR-34a Represses *Numbl* in Murine Neural Progenitor Cells and Antagonizes Neuronal Differentiation

**DOI:** 10.1371/journal.pone.0038562

**Published:** 2012-06-11

**Authors:** Sarah K. Fineberg, Poppy Datta, Colleen S. Stein, Beverly L. Davidson

**Affiliations:** 1 Department of Internal Medicine, University of Iowa, Iowa City, Iowa, United States of America; 2 Department of Molecular Physiology & Biophysics, University of Iowa, Iowa City, Iowa, United States of America; 3 Deparment of Neurology, University of Iowa, Iowa City, Iowa, United States of America; 4 Medical Scientist Training Program, University of Iowa, Iowa City, Iowa, United States of America; University of Nebraska Medical Center, United States of America

## Abstract

MicroRNA (miRNA) function is required for normal animal development, in particular in differentiation pathways from stem cell and precursor populations. In neurogenesis, it is becoming increasingly appreciated that miRNAs act at many stages to ensure proper progression. In this study we examined the role of miR-34a in neural progenitor cells (NPC) derived from murine embryonic cortex. We found that over-expression of miR-34a in NPC significantly reduced the neuron yield upon in vitro induction of differentiation. MiR-34a has several predicted targets in the Notch pathway, which operates to balance progenitor self-renewal and differentiation during cortical neurogenesis. We tested several Notch pathway players for regulation by miR-34a in undifferentiated NPC, and found that mRNA and protein levels of *Numbl*, a negative regulator of Notch signaling, as well as two downstream pro-neural genes usually blocked by Notch signaling, *NeuroD1* and *Mash1*, were diminished, while *Notch1* and *Cbf1* transcripts were enhanced by miR-34a over-expression. Using a luciferase reporter assay, we verified the *Numbl* 3′-UTR as a direct miR-34a target. Correspondingly, knock-down of endogenous miR-34a resulted in increased *Numbl*, *NeuroD1* and *Mash1*, and reduced *Notch1* transcript levels. Together these results implicate *Numbl* as a physiologically relevant target of miR-34a in NPC, allowing for enhanced Notch signaling and inhibition of neuronal differentiation. This work extends our understanding of miR-34a-mediated control of cell differentiation from cancer to mammalian nervous system development.

## Introduction

MicroRNA (miRNA) function is essential for differentiation of stem cells and organ morphogenesis [1], [2]. MiRNAs are also obligatory players in the orchestration of vertebrate nervous system development [3], [4], [5], [6]. Depletion of the miRNA-producing enzyme *Dicer* in developing mouse forebrain results in microcephaly and perinatal death [7]. Moreover, genetic ablation of dicer from mature cerebellar Purkinje [8] or forebrain neurons [9] is accompanied by prominent pathology and neuronal loss. Thus it is clear that miRNAs are critical to mature neuron integrity as well as to neural development.

MiRNA profiling studies have defined strongly expressed brain-enriched miRNAs and their regional localizations. For example, miR-124a is robustly expressed throughout the brain [10], while the miR-183-96-182 cluster is remarkable for its specific localization to neurosensory tissue [11]. Functional studies have defined neuro-developmental roles for some highly expressed miRNAs such as miR-430 in brain formation [3], miR-200 family in olfactory neurogenesis [12] and miR-124 in neuronal differentiation [13], [14].

*In situ* hybridization in zebrafish and medaka fish identified additional miRNAs relevant to vertebrate nervous system development [15], [16]. One of these, miR-34a, was robustly expressed in the developing CNS. MiR-34a shows conservation of the mature miRNA sequence in human, mouse, fish, and fly, suggesting brain developmental roles in other species. In vertebrates the miR-34 family has three members, miR-34a, b, an c, arising from two distinct loci (miR-34a from one locus and miR-34b,c from a separate locus). Bommer et al. [17] assayed mouse tissues and found miR-34a expression to be highest in the brain, while miR-34b and c were highest in lung, but low in brain. In mammalian profiling studies miR-34a level is generally low throughout the body, often escaping detection. In mammalian CNS, though miR-34a level overall is weak, several studies indicate temporal and regional enrichment. For instance, early multi-tissue microarray profiling in mouse placed miR-34a in a “late-brain development” expression cluster [18], and subsequent profiling studies in adult rodent CNS show enrichment in cerebellum [19], [20], [21], medulla oblongata, spinal cord, pons [20], and substantia nigra [21]. Sequence-based profiling in whole mouse embryos detect miR-34a at low abundance at E9.5-11.5 [22], corresponding to onset of cortical neurogenesis. Moreover, in situ hybridization on embryonic mouse brain tissue revealed miR-34a signal in the progenitor cell niche surrounding the lateral ventricle, with less expression in the cortical lamina (our unpublished observations). Together these data are consistent with roles for miR34a in both mammalian neurogenesis and mature neuron maintenance.

MiR-34 family members have been extensively studied in cancer studies where their expression has been found to impact cell cycle and apoptotic cellular pathways [23], [24]. Reduction or deletion of miR-34 is associated with higher pathologic grade and worse prognosis of many cancers, including small lung cell cancer [17], pancreatic cancer [25] and neuroblastoma [26], [27]. Conversely, forced re-expression in mouse tumor models can dramatically reduce tumor size and enhance therapies [17], [26], [27]. Furthermore, miR-34a expression is regulated by Notch and Hedgehog signaling pathways in zebrafish [28], and in tumor cells, miR34a-c are directly induced by p53 to mediate tumor suppressor functions [29]. The presence of miR-34a in developing brain and its well-demonstrated role in cell cycle control with ties to the Notch pathway, led us to investigate its role in neural progenitor cells (NPC). We show herein that modest elevation of miR-34a in murine NPC does not appreciably affect NPC cell cycling, but significantly modulates expression of Notch pathway players and counters neuronal differentiation. Moreover we show that the *Numbl* 3′UTR is targeted by miR-34a, and suggest it as a primary target in NPC.

## Methods

### Vectors/Plasmids

Figure S1A illustrates plasmids used in this study. Shuttle plasmids used for generating replication-deficient feline immunodeficiency virus (FIV)-based lentiviral vectors, and methods for vector production are described in more detail in a previous report [30]. For lentiviral vectors, the genomic murine miR-34a sequence (NR_029751.1) was cloned into the 3′UTR of green fluorescent protein (GFP), or was cloned into a U6 promoter-miRNA-polyT cassette, of the appropriate shuttle plasmid. Viral vectors were produced by co-transfection of shuttle plasmid, packaging plasmid, and VSV-G envelope plasmid in producing cells, according to standard protocols in the University of Iowa Gene Transfer Vector Core (http://www.uiowa.edu/~gene/). Vectors were titered at 1–6 X 10^8^ transducing units/ml. For construction of Psicheck2™-34T, forward (5'-acaaccagctaagacactgccagcggccgctat-3') and reverse (5'-tggcagtgtcttagctggttgtatagcggccgc-3') primers were annealed and extended by PCR to generate 5'-acaaccagctaagacactgccagcggccgctatacaaccagctaagacactgcca-3' containing two perfect miR-34a target sites (underlined) with complete complementary to mature miR-34a.

This product was cloned into pCR™2.1-TOPO (Invitrogen, Carlsbad, CA) and sub-cloned into the 3'UTR of firefly luciferase in Psicheck2™ (Promega Corporation, Madison, WI). Psicheck2™ with *Numbl* target sites was generated by cloning the entire 3'UTR of murine *Numbl* (bp1993–2733 of NM_010950) into the 3′UTR of firefly luciferase in Psicheck2™. To eliminate *Numbl* miR-34 target sites, 4 bases within the seeds were mutated using QuikChange mutagenesis (Agilent Technologies, Santa Clara, CA), changing 5'-ca**ctgc**c-3' to 5'-ca**gacg**c-3'.

### Animal Husbandry

C57Bl6/J mice were purchased from The Jackson Laboratory (Bar Harbor, Maine) or bred in-house. All experiments were approved by the University of Iowa Animal Care and Use Committee, and procedures were performed in accordance with those guidelines. Animals were housed in the University of Iowa Animal Care facilities, and maintained on a 12h:12h light:dark cycle with free access to food and water.

### NPC Preparation

Timed matings were set up with one male and one to three females co-housed in the male’s home cage from one afternoon until the next morning. Embryonic day 0.5 (E0.5) was defined as noon on the day the animals were separated. To prepare NPC cultures, mouse embryos were collected at E11.5, E13.5, or E15.5 from pregnant dams. Dams were over-anaesthetized with 0.2 ml/g of a 1% ketamine/10% xylazine mixture followed by cervical dislocation. Embryos were rapidly removed, rinsed in PBS with 2% glucose (PBSG) and decapitated. Brains were removed, and whole cortex was isolated and diced, then triturated in PBSG using cotton-stuffed sterile glass Pasteur pipettes. The solution was transferred to 15-ml falcon tubes and diced tissue was allowed to settle. The supernatant was transferred to a fresh tube. NPC maintenance media (Stem Cell Technologies, Vancouver, Canada) was added to the remaining tissue pieces, which were again triturated to dissociate more cells, and the supernatant was transferred. This procedure was repeated 5 times, then the remaining tissue pieces were discarded, and the supernatant was centrifuged at 800 rpm at 4°C for 5 min. A cell pellet was easily discerned, and the clear supernatant was discarded and replaced with ∼30 ml fresh maintenance media. Cells were seeded into T-150 filter flasks at a density of 2 X10^6^ cells/100 ml for suspension growth in tissue culture incubators at 37°C and 5% CO_2_. Cells were allowed to divide for ∼5 days, forming medium-sized neurospheres. They were then used for experiments or passaged by gentle trituration and re-seeding in fresh maintenance media at lower density. All cells used for experiments had been passaged six or fewer times.

### Lentiviral-mediated Transduction of NPC

For NPC infections, neurospheres were gently dissociated by trituration and 1 X 10^6^ cells/well were plated in 12-well tissue culture plates in 1 ml maintenance media and virus was added at a multiplicity of infection (MOI) of 3. Cells were transferred to T-25 flasks with 8 ml of NPC maintenance media 18–24 hours later and cultured for 3 days to medium-sized neurospheres. Neurospheres infected with FIVneo^r^U634a or FIVneo^r^U6cntrl lentiviral vectors were selected for neomycin resistance by culturing in maintenance media containing 500 µg/ml G418 (Invitrogen) for 3 days, then surviving cells were used directly in experiments.

### NPC Differentiation

For differentiation experiments, neurospheres were dissociated using HyQtase (HyClone, Logan, UT) for 15 min at 37°C followed by gentle mechanical trituration. They were then centrifuged at 1000 xg at 4°C for 5 min, then resuspended in NPC differentiation media (Stem Cell Technologies) or IGF media (Hyclone DMEM/F12 with addition of 1% penicillin/streptomycin, 1% l-glutamine, 1% FBS, 0.3% dextrose, 20 ng/ml insulin-like growth factor-1 (IGF-1)) (Sigma, St. Louis, MO) and seeded into poly-L-ornithine-coated tissue culture plates or glass chamber slides.

### Secondary Neurosphere Formation Assay

E15.5-derived NPC were transduced with FIVGFP or FIVGFP34a vectors as described above, and were mechanically dissociated and plated at equal density in 96-well plates. After seven days, the number of neurospheres per plate and the average neurosphere size were measured using Image J software.

### BrdU/PI Flow Cytometric Analysis of Cell Cycle

E15.5-derived NPC were transduced with FIVneo^r^U634a or FIVneo^r^U6cntrl, and selected in G418. 5-bromo-2′-deoxyuridine/propidium iodide (BrdU/PI) cell cycle analysis (similar to Menon et al. [31]) was performed on proliferating cells (2 and 3 days after seeding in maintenance media) and differentiating cells at 3, 6, or 10 h after seeding dissociated cells into polyornithine-coated dishes in differentiation media. Cells were pulsed with BrdU (20 µM, Sigma) for 1 h prior to harvest. At harvest, cells were dissociated with HyQtase, pelleted and resuspended in 400 µl ice-cold PBS. Cells were fixed by addition of 3.6 ml ice-cold 95% ethanol while vortexing, and stored overnight at 4°C. Fixed cells were washed in PBS-TB (PBS with 0.2% tween-20 and 0.1% BSA), and resuspended in 2N HCL with 0.5% Triton X-100 and 0.2 mg/ml pepsin (to release cell nuclei) for 30 min at room temperature with gentle vortexing every 5 min. Three volumes of 0.1 M sodium tetraborate were added, nuclei pelleted and resuspended in PBS-TB and stained with 1/500 anti-BrdU (G3G4, University of Iowa DSHB, Iowa City, IA) for 1 h followed by 1/1500 Alexa Fluor-488-conjugated goat anti-mouse IgG (Invitrogen) for 30 min at room temperature. Washed nuclei were resuspended in PBS containing 20 µg/ml PI, 200 µg/ml RNaseA and 0.05% tween-20, and after 30 min incubation at room temperature were passed through 70-µm mesh and collected on a Becton Dickinson FACScan in the University of Iowa Flow Cytometry Facility (http://www.healthcare.uiowa.edu/corefacilities/flowcytometry/). Ten thousand to 15,000 single-nuclei events were acquired and analyzed as dual-parameter linear PI-A vs log-Alexa 488 dot-plots. Cellquest Pro software was used to determine cell proportions in quadrants (UL, UR, LL, LR), with total BrdU positive (UL+UR) representing S-phase cells, and LL and LR representing G1 and G2/M phases respectively.

### Immunofluorescent Staining and Cell Counts

NPC were transduced with FIVneo^r^U634a or FIVneo^r^U6cntrl, selected in G418, and differentiated as described above. After five days, cells were fixed in 4% paraformaldehyde for 20 min at 25°C, then blocked for 30 min in 0.05% Triton-X-100, 5% goat serum, PBS. Primary antibody incubation proceeded at 4°C overnight. Anti- microtubule-associated protein type 2 (anti-MAP2) (1∶200, Sigma) was used to detect neurons and anti- glial fibrillary acidic protein-Cy3 (anti-GFAP-Cy3) (1∶5000, Sigma) was used to detect glia. Secondary antibody incubation was at 25°C for 1 hour. Fluorescent images were captured under identical conditions for each fluorochrome. Neurons were identified by bright MAP2 staining in the cell body and within thin projections, and negative GFAP staining. Glial cells were identified as GFAP-bright, MAP2-negative cells and the proportion of neurons and glia were determined from 800 total cell counts per culture.

### Measurement of Transcript Levels

Total RNA was collected from NPC or HEK293 cells using Trizol (Invitrogen) according to the manufacturer’s instructions. RNA quantity and quality was measured using a nanodrop ND-1000 (Thermo Fisher Scientific, Wilmington, DE). Reverse transcription (RT) reactions were performed using the High Capacity cDNA Archive kit (Applied Biosystems (ABI), Foster City, CA), with random primers for mRNAs or specific primers for miR-34a (miR assay ID 426, ABI). Total 140 ng RNA (for miRNA) and 200 ng RNA (for mRNA) were used in 50-µl RT reactions. Gene expression primer-probe sets were purchased from ABI: *Numbl* (assay ID: Mm00477931), *Notch1* (assay ID: Mm00435245), *Cbf1* (assay ID: Mm01217626), *NeuroD1* (assay ID: Mm01200117), *Mash1* (assay ID: Mm03058063) and *Rpl34* (assay ID: Mm01321800). cDNA samples were used at a final dilution of 1/45 in 20-µl quantitative PCR (qPCR) reactions (Prism 7900HT and TaqMan 2× Universal Master Mix; ABI). Transcript levels were determined by interpolation off of standard curves generated from known positive samples, and *Rpl34* was use as an endogenous control for normalization.

### Anti-miR Inhibition of miR-34a

100 nM or 200 nM of anti-miR miRNA inhibitor (AM11030, ABI)) or scrambled negative control (ABI) were used for inhibition of miR-34a in NPC. The anti-miR was added to singly dissociated cells in suspension and electroporation was performed using the Neon Transfection System (Invitrogen). Total RNA was harvested 72 h later with TRIzol (Invitrogen), and RT-qPCR performed as described above.

### Western Blot

NPC transduced with FIVneorU634a or FIVneorU6cntrl were harvested in RIPA lysis buffer containing 1X protease inhibitor (Pierce Biotechnology, Rockford, IL) using standard techniques, and protein concentrations were determined by DC Protein Assay (Bio-Rad, Hercules, CA). Samples were reduced and separated by SDS-PAGE on 4%–10% acrylamide gels and transferred to Immobilon PVDF transfer membranes (Millipore, Billerica, MA). The primary antibodies used were as follows: Numbl (1∶1000, Abcam, Cambridge, MA), NeuroD1 (1∶1000, Millipore), Mash1 (1∶200, BD Biosciences Pharmingen, San Diego, CA), β-actin (1∶5000, Sigma), and β-catenin (1∶5000 Abcam). Blots were developed using ECL Plus Western Blotting Detection System (GE Healthcare, Pittsburg, PA), images captured using VersaDoc imaging system (Bio-Rad) and quantified by densitometric analyisis using Quantity One software (Bio-Rad). Band densities were normalized to house-keeping (β-actin or β-catenin) band densities in the same lanes.

### Psicheck2™ Luciferase Assays

For luciferase assays, lentiviral vector shuttle plasmid (control or miR-34a-expressing) and appropriate Psicheck2™ luciferase reporter construct were co-transfected into HEK293 cells at a ratio of 8 ng:1ng using lipofectamine 2000 (Invitrogen) according to the manufacturer’s instructions. Lysates were collected 18–24 h after transfection and luciferase activity was measured using the dual-luciferase kit (Promega) according to manufacturer’s instructions, using a Monolight 3010C Luminometer (BD Biosciences Pharmingen). Firefly luciferase activity was normalized to renilla luciferase activity.

### Statistics

Unless otherwise indicated in the legend, data are plotted as mean ± standard deviation (SD) of replicate experiments. Statistical differences between test and control groups were determined by Mann-Whitney rank sum test (sphere size and BrdU/PI flow cytometry) or Student's *t* test.

**Figure 1:**
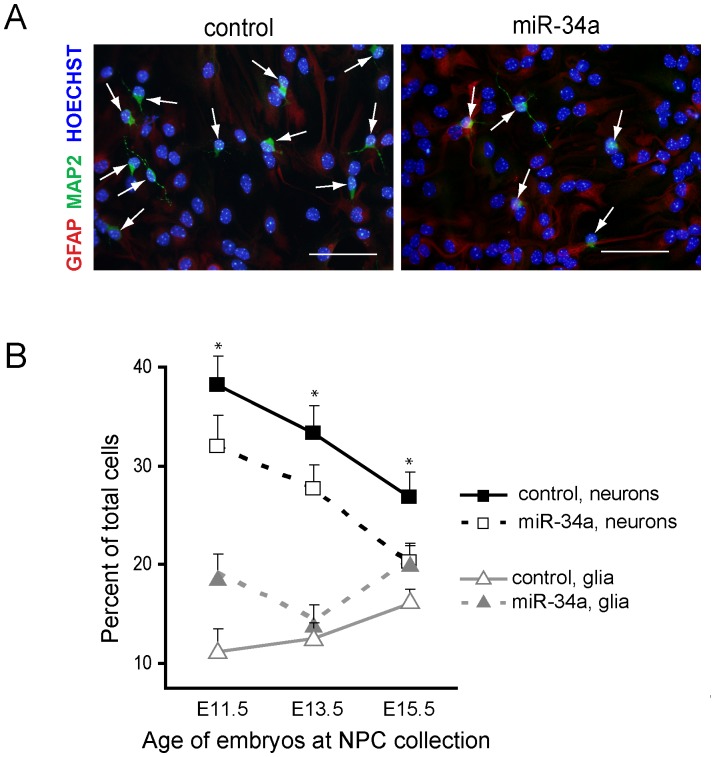
MiR-34a over-expression in NPC reduces neuron yield upon differentiation. NPC generated from E11.5, E13.5 and E15.5 cortices were transduced with FIVneo^r^U6cntrl (control) or FIVneo^r^U634a (miR-34a) and differentiated for 5 days as described in Methods. Neurons and glia were identified by MAP2 and GFAP immunofluorescence labeling. *A)* Representative image after differentiation of E15.5 NPC, showing MAP2 positive neurons (green), GFAP positive astrocytes (red), and cell nuclei (hoechst stain, blue). Arrows indicate neurons. Scale bar 100-µm. *B)* The percentage of neurons and glia were determined for each culture. Data points represent the mean ± SD percentage of neurons or glia in cultures. *p<0.05, for neurons in miR-34 over-expressing compared to control.

**Figure 2:**
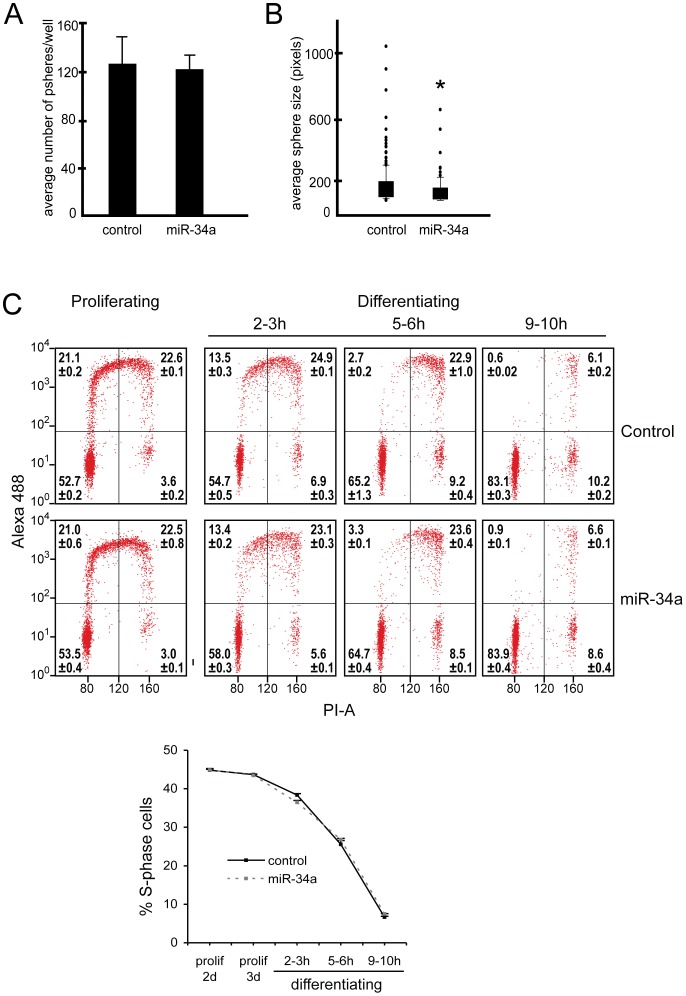
Cell cycle analysis. NPC established from E15.5 were infected with miR-34a-expressing or control lentiviral vectors and proliferation parameters assessed. *A* and *B)* In non-differentiation conditions, NPC transduced with FIVGFP (control) or FIVGFP34a (miR-34a) were dissociated and plated at low density and one week later neurospheres were counted (*A*, bar graphs) and sphere size measured and (*B*, box plots). For neurosphere counts, bars represent the mean ± SD of three independent cultures. For sphere size, data was combined from three independent cultures. Boxes encompass 25th-75th percentile and whiskers mark 10th and 90th percentiles. *p<0.001, for miR-34a over-expressing compared to control. *C)* NPC transduced with FIVneo^r^U6cntrl (control) or FIVneo^r^U634a (miR-34a) were BrdU-pulsed for 1 h under proliferating conditions (2 or 3 days after passage), or were seeded into differentiation media and BrdU-pulsed at 2–3 h, 5–6 h, or 9–10 h post-seeding. Cells were collected directly after pulse and analyzed for BrdU incorporation (A488 intensity) and DNA content (PI-A). Representative dot-plots are shown, and quadrant statistics for 3 replicate experiments are displayed on the dot-plots (mean ±SEM). The line graph illustrates the proportion of cells in S-phase (BrdU positive cells, mean ±SEM of the three experiments). No significant differences in cell cycle distribution were determined (*p*>0.05 for miR-34a vs control).

## Results

To test our prediction that miR-34a functions in NPC, we generated NPC from mouse embryonic cortex, and examined the effects of miR-34a over-expression or reduction. For over-expression, we used replication-deficient lentiviral vectors (Fig S1a) to stably introduce a miR34a expression cassette, and for knock-down of endogenous miR34a, we transfected with anti-miR miRNA inhibitors. We first tested our lentiviral constructs for expression of functional miR-34a by using the Psicheck2™ luciferase reporter system. HEK293 cells were co-transfected with the lentiviral shuttle plasmid (miR-34a or control) and the Psicheck2™ plasmid encoding firefly luciferase fused to miR-34a perfect target sites (Psicheck2™-34T). The miR-34a expression vectors repressed firefly luciferase activity by 50–75% (Fig. S1b), verifying miR-34a expression and processing to the mature functional form. The lentiviral constructs were subsequently used for viral vector production. We verified the activities of lentiviral vectors and anti-miR oligonucleotides in our NPC: transduction with miR-34a-expressing lentiviral vectors increased miR-34a levels 1.6–4.5 fold relative to control-transduced cultures, and transfection with anti-miR lowered endogenous miR-34a levels to approximately 20–30% of control (Fig S1c).

**Figure 3:**
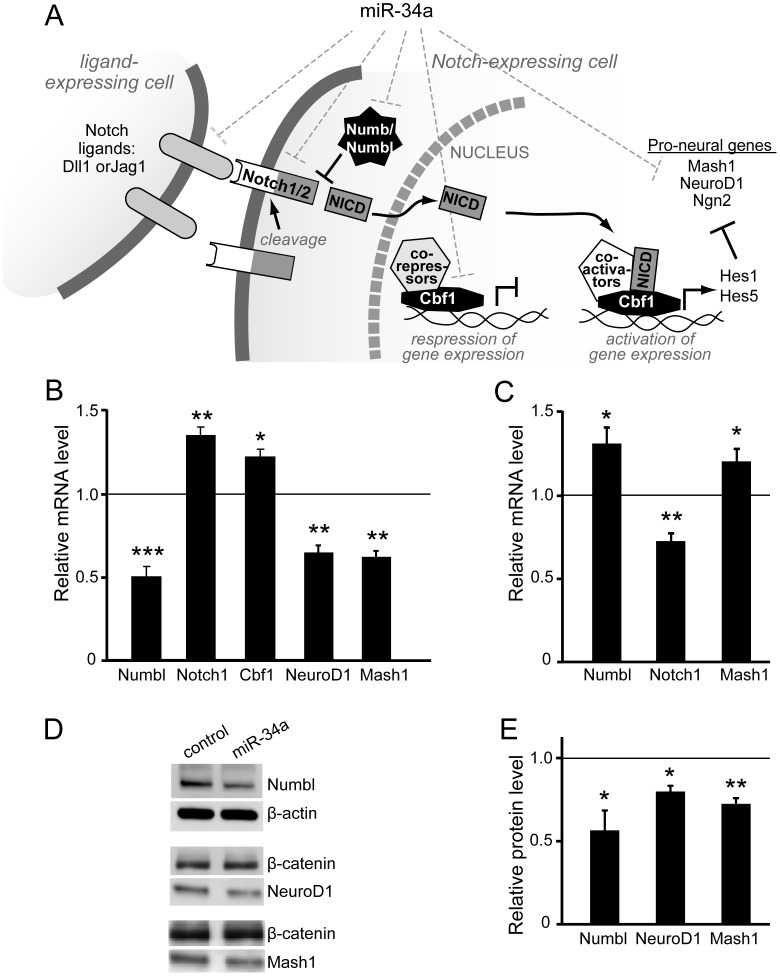
MiR-34a potentiates Notch pathway activity. *A)* Schematic depicting the Notch pathway. Ligand binding initiates Notch1 cleavage, with release the intracellular domain (NICD). NICD translocates to the nucleus and binds to Cbf1, displacing transcriptional repressors and promoting activator binding for induction of Hes1 and Hes5, which in-turn repress the expression of pro-neural genes. Numb and Numbl directly inhibit Notch signaling. Dotted lines indicate predicted Notch pathway targets: Dll1, Jag1, Notch1, Notch2, Numbl, Cbf1 and Mash1. *B)* E15.5-derived NPC were transduced with FIVneo^r^U6cntrl (control) or FIVneo^r^U634a (miR-34a) lentiviral vectors and RNA was collected and transcript levels determined by RT-qPCR for Notch pathway genes. Results show transcript abundance in miR-34a over-expressing relative to control NPC (control set to 1, horizontal line). Bars represent the mean ± SD of three cultures. *p<0.05, **p<0.01, ***p<0.005, relative to control. *C)* NPC generated from E15.5 were transfected with 100 nM anti-miR oligonulceotides targeting miR-34a or negative control oligonucleotides. RNA was collected 72 h post-transfection and relative transcript abundance determined from three separate experiments as described in *B*. *p<0.05, **p<0.01, relative to control. *D* and *E)* NPC were generated and infected as described in *B*, and cell lysates analyzed by Western blot. *D)* Representative immunoblots for Numbl, NeuroD1, and Mash1. E) Protein abundance was determined by densitometric analysis of immunoblot bands and normalization to a housekeeping protein (either β-actin or β-catenin). Results show mean ± SD relative to control NPC (control set to 1, horizontal line) of three separate cultures. *p<0.05, **p<0.01, relative to control.

**Figure 4:**
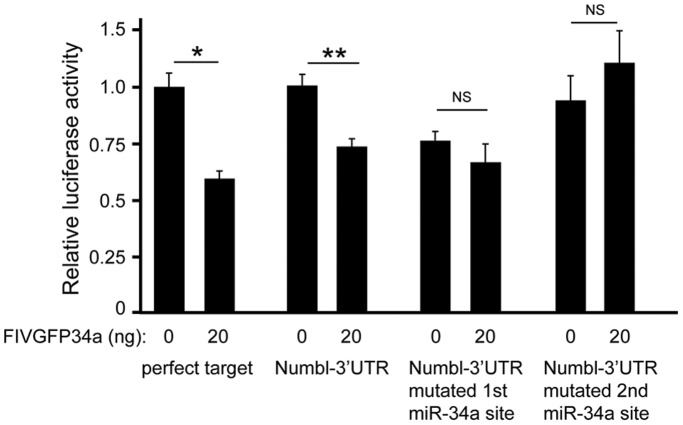
Numbl is a direct target of miR-34a. HEK293 cells were co-transfected with the miR-34a-expressing shuttle plasmid (FIVGFP34a) and Psicheck2™-34T (with two miR-34a perfect target sites), or Psicheck2™ with the *Numbl* 3'UTR, or the *Numbl* 3'UTR mutated in the first or second miR-34 binding site, cloned 3' of the firefly luciferase coding region. Luciferase assay was used to quantify miR-34a-dependent repression of firefly luciferase. Bars show the mean ± SD of 2 (perfect target) or 3 experiments. *p<0.05, **p<0.005, luciferase activity compared to control. NS, not significant.

Next, we assessed the effect of miR34a over-expression on NPC differentiation. NPC were established from 3 time points during cortical neurogenesis- E11.5, E13.5, and E15.5. We effected gain of miR-34a in cultured NPC by transducing with FIVneo^r^U634a. Control NPC were transduced with FIVneo^r^U6cntrl. After selecting in G418, NPC were plated in differentiation-promoting conditions as detailed in Methods. Five days later, neurons and glial cells were identified by immunofluorescent staining and morphological criteria (Fig. 1a). A substantial difference in cell counts indicated that this modest over-expression of miR-34a had a potent effect on neurogenesis; neuron production from NPC initiated at all three time points was significantly repressed relative to control cultures (Fig. 1b).

Based on enhanced glial cell production at E11.5 and E15.5 (Fig. 1b), cellular fate shift may account for some of the neuron loss in these cultures. However, at E13.5, neuron production was decreased while the glial population was similar to control cultures. We therefore tested a role for slowed cell cycling in NPC transduced with miR-34a-encoding virus. After chemical dissociation, miR-34a- or control-infected NPC were re-plated in proliferation or differentiation conditions. Under proliferation conditions, miR-34a over-expression did not change secondary neurosphere number (Fig. 2a). Secondary neurosphere size, however, was slightly but significantly reduced (Fig. 2b, median size was 136 vs. 160 pixels). For cell cycle analysis, cells received a 1 h pulse with the thymidine analog BrdU, and cell nuclei were subsequently extracted and analyzed by flow cytometry for BrdU incorporation and DNA content. The distribution of cells in S-phase (BrdU positive), G1 (BrdU negative, 2N DNA), and G2/M (BrdU negative, 4N DNA), was not significantly affected by miR-34a over-expression in either proliferating or differentiating (3, 6, or 10 h post-differentiation) NPC (Fig. 2c). These results indicate that in our system, unlike cancer cell lines, miR-34a over-expression does not promote cell cycle exit, and therefore progenitor depletion via premature cell cycle exit does not explain reduced neuron counts upon differentiation.

We next turned to available target prediction software for indication that miR-34a might work within a molecular pathway that regulates neural differentiation. By querying *targetscan.org* we noticed that several genes in the Notch pathway [32] are predicted murine targets of miR-34, including the anti-differentiation factors, *Notch1, Notch2,* and *Cbf1(Rbpj),* the pro-differentiation factors *Numbl* and *Mash1(Ascl1)*, and the Notch ligands *Jag1 and Dll1*. Our schematic (Fig. 3a) outlines relevant components of the canonical Notch pathway; ligand binding triggers Notch cleavage and translocation of the Notch intracellular domain to the nucleus where it associates with Cbf1 to activate *Hes1*/*Hes5* transcription, which in-turn repress expression of pro-neural genes *NeuroD1*, *neurog2* and *Mash1*. Numb/Numbl directly block Notch to prevent signaling. Depending on which predicted targets are real and physiologically relevant during neurogenesis, miR-34a could promote or antagonize differentiation.

To determine the ability of miR-34a to regulate Notch pathway players in NPC, we transduced NPC with FIVneo^r^U634a or FIVneorU6cntrl vectors, selected the transduced NPC by culturing in G418, and assessed mRNA levels of *Numbl*, *Notch1*, *Cbf1*, *NeuroD1*, and *Mash1*. Interestingly we found that transcript levels of *Notch1* and *Cbf1* were elevated in the context of miR-34a over-expression (Fig 3b). In contrast, mRNA levels of *Numbl*, *NeuroD1*, and *Mash1* were repressed in miR-34a-overexpressing NPC (Fig 3b). Consistent with this, down-regulation of endogenous miR-34a by anti-miR treatment led to decrease in *Notch1* and increases in *Numbl* and *Mash1* mRNA levels (Fig 3c). Densitometric analysis of immunoblots indicated that changes in protein levels upon miR34a over-expression were in agreement with transcript data; protein levels of Numbl, NeuroD1 and Mash1 were reduced, while Notch1 was elevated. (Fig. 3d,e). Together, these observations provide strong evidence that miR-34a functions to repress negative signals within the Notch pathway and potentiate Notch1 signaling in NPC, thereby antagonizing neuronal differentiation.

Decline in *Numbl* transcript and protein quantities prompted us to evaluate *Numbl* as a direct miR-34a target. We therefore cloned the murine *Numbl* 3′UTR, which contains two predicted target sites for miR-34a, into the Psicheck2™ reporter plasmid and used site-directed mutagenesis to separately ablate each target site. We subsequently challenged these reporters with miR-34a over-expression in HEK293 cells. Indeed, miR-34a mediated significant repression of the luciferase reporter fused to the native *Numbl* 3'UTR, while mutation of the either target site abrogated repression (Fig. 4), suggesting that the two target sites likely cooperate to mediate miR-34a repression of *Numbl*. Collectively our results show that miR34a operates as a regulator within the Notch pathway in NPC, where it directly targets *Numbl* transcripts.

## Discussion

In this study we generated NPC with moderate over-expression of miR-34a, and examined the effects on subsequent differentiation. Intriguingly, we found that neuronal output from these NPC was substantially impaired. We further determined that miR-34a expression influenced transcript and protein abundance of several Notch pathway players in NPC, thus implicating miR34a as a regulatory component of the Notch pathway. Moreover, we provide novel evidence that *Numbl* is a direct miR-34a target. Together these results suggest that miR-34a antagonizes neuronal differentiation in progenitor cells at least in part by direct repression of *Numbl*.

In tumor cells, miR34a-c act as p53 effectors, mediating down-regulation of cell-cycling gene programs and promoting apoptosis [24], [25]. We thus reasoned that reduced neuron numbers in our differentiated NPC cultures could reflect changes in the progenitor cell cycle. Cell cycle progression in NPC is intimately involved with the state of differentiation. The length of the G1 phase of the cell cycle increases during neurogenesis and is thought to facilitate protein synthesis and integration of signals needed for neuronal specification and differentiation [33], [34]. Upon examining cell cycling parameters however, we found that our modest over-expression of miR-34a did not significantly change the percent self-renewing neural stem cells (measured by sphere number) or percent cells in S, G1, G2/M (determined by BrdU incorporation and DNA content). This coincides with minimal effects of miR-34a over-expression in an astrocyte cell line [35], but contrasts with the profound anti-proliferative effects of mir-34a over-expression in various tumor cells [23]. When we exposed NPC to differentiation conditions, control- and miR-34a-transduced NPC showed very similar patterns of progressive decline in BrdU incorporation and increase in the G1 population from 3 to 10 h. Thus our data do not support accelerated cell cycle exit as an explanation for the reduced neuron output after differentiation.

Numb, Numbl and Notch1 are critical players in the complex regulation of cortical neurogenesis, balancing progenitor self-renewal, proliferation and differentiation [36], [37], [38]. We considered that miR-34a might influence neurogenesis via regulation of protein expression in the Notch pathway. Interestingly we found that miR-34a over-expression altered transcript and protein levels of several players, in a pro-Notch signaling direction. We also confirmed the *Numbl* 3'UTR as a direct target of miR-34a. Numbl and Numb are highly related, though Numbl is less characterized. They have many overlapping functions, and share the ability to bind Notch to inhibit Notch signaling [36], [39], [40]. Asymmetrical cell division with unequal inheritance of Numb/Numbl dictates relative Notch1 signaling and is a key mechanism to regulate the timing of neurogenesis [41]. Interestingly, Numbl has recently been implicated in promoting adult neural stem cell differentiation; by acting directly on *Numbl*, miR-184 mitigated neuronal maturation [42]. Thus, Numbl appears to be a critical player in neuronal differentiation pathways and a miRNA target in neuronal progenitor cells.

Notch1 is ubiquitously expressed across tissues and cells, and the cellular outcome of Notch1 signaling can be pro- or anti-proliferative depending on the cell type and the context of additional intrinsic and extrinsic cues [38]. During mammalian cortical neurogenesis, Notch1 signaling is believed to delay differentiation, consistent with its transcriptional repression of pro-neural genes such as *Mash1* and *NeuroD1* [43]. Numbl is postulated to augment terminal differentiation by repressing residual Notch1 activity in post-mitotic neuronal precursors that have reached the cortical plate [36]. We observed enhanced *Notch1* and reduced *Mash1* and *NeuroD1* transcript and protein levels with miR-34a over-expression, indicating that delayed progression to terminal differentiation may be an important contributing factor to diminished neuron counts achieved post-differentiation. Lastly, Notch1 signaling to precursors after late divisions plays an instructive role in gliogenesis [44], [45], and this could explain the increased astrocyte counts encountered after differentiation of some of our miR34a-overexpressing NPC cultures.

It was somewhat surprising that *Notch1* was not repressed by miR34a over-expression in NPC. The *Notch1* 3'UTR harbors predicted miR-34 binding sites, and recent studies in cancer cell systems show that miR-34a directly represses *Notch1/2*, to slow proliferation [46] or invasiveness [47]. MiR-34a repression of *Numbl* rather than *Notch1* in our NPC may relate to transcript abundance and differential miR-34a binding affinities. It is also possible miR-34a indirectly enhances *Notch1* expression through an un-described pathway, and that within NPC, this indirect enhancement dominates over direct repression.

Interestingly, recent studies [48], [49] showed that endogenous miR-34a expression rose dramatically during in vitro differentiation of neural stem cells, to peak on day 6, and that miR-34a over-expression during differentiation enhanced neuronal output and neurite length. Their findings in combination with ours suggest that miR-34a may counter differentiation in progenitors, but that as differentiation proceeds miR34a escalates to potentiate maturation and stabilize neuronal status. Thus miR-34a may act on distinct targets in temporal fashion in accordance with expression levels and target availability, to regulate progenitor self-renewal and fine-tune differentiation kinetics.

Numb has recently been shown to modulate p53 levels by shielding it from ubiquitination and degradation [50]. Moreover, p53 has been shown to induce expression of Notch1 [51] in addition to miR-34a [25] in cancer cells. In the context of NPC, we have revealed *Numbl* as a direct miR-34a target, possibly operating within a p53-Notch interconnected pathway to balance proliferation and differentiation. Continued exploration will address important questions regarding miR34a regulation of other predicted targets within the p53-Notch axis and potential impact on CNS developmental programs.

## Supporting Information

Figure S1 **Verification of plasmids, lentiviral vectors and anti-miR.** A) Depictions of constructs used in this study. FIV (feline immunodeficiency virus) shuttle plasmids were used to produce replication-deficient FIV-based lentiviral vectors, and in some experiments were used directly in transfections as expression plasmids. Abbreviations in construct names: CMV, cytomegalovirus immediate early promoter; GFP, green fluorescent protein; U6, U6 promoter; LTR, long terminal repeat region of FIV; neor, neomycin resistance gene; 34a, miR-34a gene sequence; cntrl, scrambled control sequence; 34T, perfect target sequence for mature miR-34a binding; Rluc, renilla luciferase; FFluc, firefly luciferase; pA, polyadenylation sequence. B) Shuttle plasmids were tested for expression of functional miR-34a by co-transfection with Psicheck2TM-34T in HEK293 cells and measurement of luciferase activity. Bars show the mean ± SD of three to five experiments. *p<0.0001 for luciferase activity in cultures with miR-34a-expressing plasmids (FIVGFP-34a or FIVGFPU634a) compared to control plasmid (FIVGFP). C) Lentiviral vectors and anti-miR34a oligonucleotides were tested for function in NPC. NPC were transduced with the indicated miR-34-expressing lentiviral vector its control counterpart. RNA was isolated and relative levels of mature miR-34a quantified by RT-qPCR, and are expressed relative to the respective control (control set to 1, horizontal bar). To test anti-miR, E15.5-derived NPC were transfected with anti-miR34a oligonucleotides or negative control oligonucleotides. RNA was isolated after 72 h and mature miR-34a was quantified by RT-qPCR, and is expressed relative to the respective control (control set to 1, horizontal bar). Results show the mean ± SD of three or four experiments. *p<0.05, **p≤0.0001 compared to control.
(TIF)
