# Supplementary figures and images for: MiR-34a Represses Numbl in Murine Neural Progenitor Cells and Antagonizes Neuronal Differentiation
Source: PLoS One. 2012 Jun 11;7(6):e38562. doi: 10.1371/journal.pone.0038562 (PMC3372529; doi:10.1371/journal.pone.0038562)

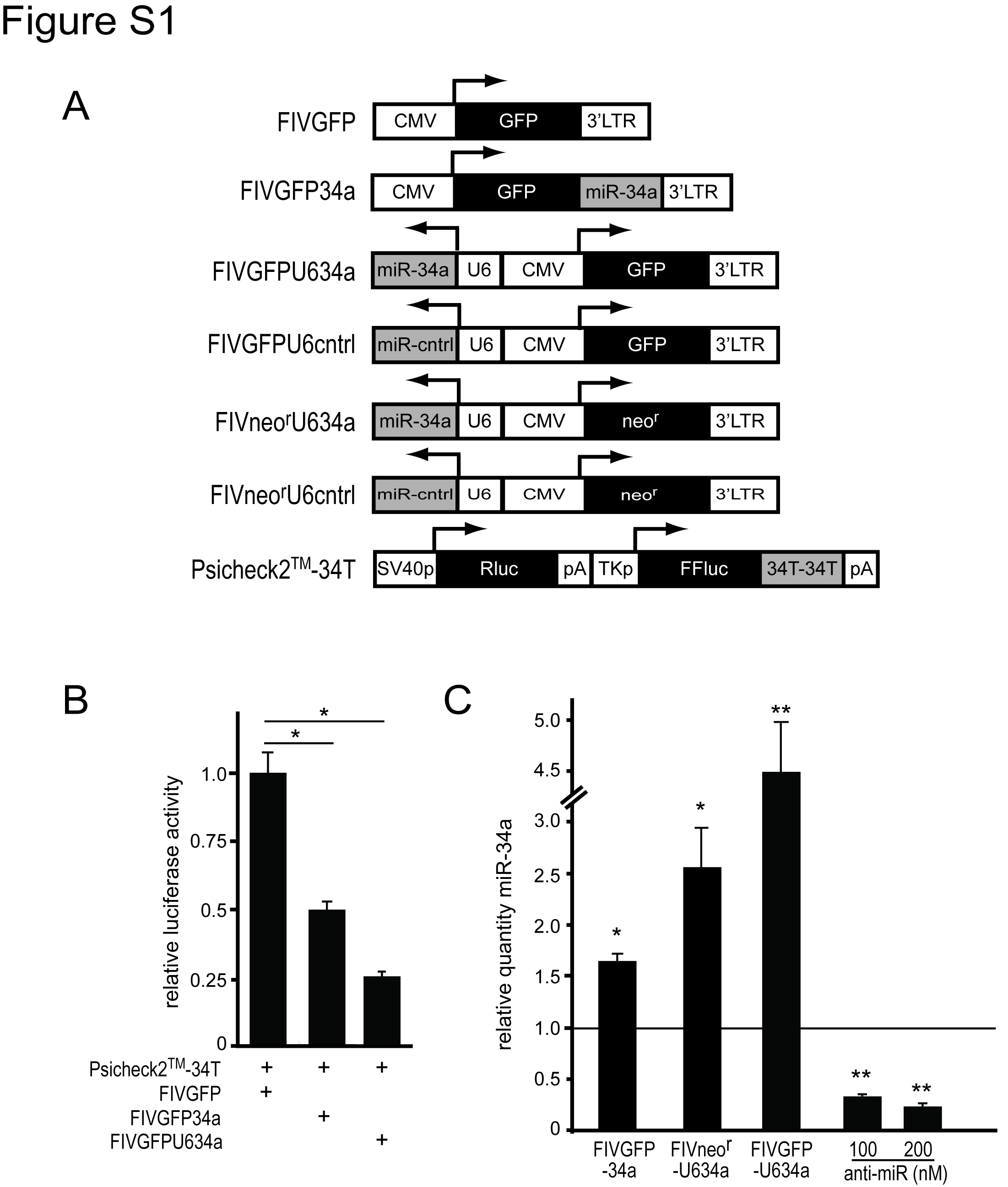

Supplement: Figure S1 — Verification of plasmids, lentiviral vectors and anti-miR. A) Depictions of constructs used in this study. FIV (feline immunodeficiency virus) shuttle plasmids were used to produce replication-deficient FIV-based lentiviral vectors, and in some experiments were used directly in transfections as expression plasmids. Abbreviations in construct names: CMV, cytomegalovirus immediate early promoter; GFP, green fluorescent protein; U6, U6 promoter; LTR, long terminal repeat region of FIV; neor, neomycin resistance gene; 34a, miR-34a gene sequence; cntrl, scrambled control sequence; 34T, perfect target sequence for mature miR-34a binding; Rluc, renilla luciferase; FFluc, firefly luciferase; pA, polyadenylation sequence. B) Shuttle plasmids were tested for expression of functional miR-34a by co-transfection with Psicheck2TM-34T in HEK293 cells and measurement of luciferase activity. Bars show the mean ± SD of three to five experiments. *p<0.0001 for luciferase activity in cultures with miR-34a-expressing plasmids (FIVGFP-34a or FIVGFPU634a) compared to control plasmid (FIVGFP). C) Lentiviral vectors and anti-miR34a oligonucleotides were tested for function in NPC. NPC were transduced with the indicated miR-34-expressing lentiviral vector its control counterpart. RNA was isolated and relative levels of mature miR-34a quantified by RT-qPCR, and are expressed relative to the respective control (control set to 1, horizontal bar). To test anti-miR, E15.5-derived NPC were transfected with anti-miR34a oligonucleotides or negative control oligonucleotides. RNA was isolated after 72 h and mature miR-34a was quantified by RT-qPCR, and is expressed relative to the respective control (control set to 1, horizontal bar). Results show the mean ± SD of three or four experiments. *p<0.05, **p≤0.0001 compared to control. (TIF) [file pone.0038562.s001.tif]
